# Plant Disease Management: Leveraging on the Plant-Microbe-Soil Interface in the Biorational Use of Organic Amendments

**DOI:** 10.3389/fpls.2021.700507

**Published:** 2021-07-30

**Authors:** Akinlolu Olalekan Akanmu, Olubukola Oluranti Babalola, Vittorio Venturi, Modupe Stella Ayilara, Bartholomew Saanu Adeleke, Adenike Eunice Amoo, Ayodele Adegboyega Sobowale, Ayomide Emmanuel Fadiji, Bernard R. Glick

**Affiliations:** ^1^Food Security and Safety Niche, Faculty of Natural and Agricultural Sciences, North-West University, Mmabatho, South Africa; ^2^Bacteriology Group, International Centre for Genetic Engineering and Biotechnology, Area Science Park, Trieste, Italy; ^3^Department of Botany, Faculty of Science, University of Ibadan, Ibadan, Nigeria; ^4^Department of Biology, University of Waterloo, Waterloo, ON, Canada

**Keywords:** modern agriculture, disease suppression, quorum formation, rhizosphere, induced systemic resistance, systemic acquired resistance

## Abstract

Agriculture is faced with many challenges including loss of biodiversity, chemical contamination of soils, and plant pests and diseases, all of which can directly compromise plant productivity and health. In addition, inadequate agricultural practices which characterize conventional farming play a contributory role in the disruption of the plant-microbe and soil-plant interactions. This review discusses the role of organic amendments in the restoration of soil health and plant disease management. While the use of organic amendments in agriculture is not new, there is a lack of knowledge regarding its safe and proper deployment. Hence, a biorational approach of organic amendment use to achieve sustainable agricultural practices entails the deployment of botanicals, microbial pesticides, and organic minerals as organic amendments for attaining plant fitness and disease suppression. Here, the focus is on the rhizosphere microbial communities. The role of organic amendments in stimulating beneficial microbe quorum formation related to the host-plant-pathogen interactions, and its role in facilitating induced systemic resistance and systemic-acquired resistance against diseases was evaluated. Organic amendments serve as soil conditioners, and their mechanism of action needs to be further elaborated to ensure food safety.

## Introduction

Modern agriculture is a rapidly changing technology that improves the efficiency of agricultural activities while minimizing the use of limited resources, such as water, energy, and space to satisfy the world’s growing food demand. As such, the practice shows tremendous improvements in crop yield and food safety compared to conventional agriculture which is described by its uniformity, efficiency, and maximization of resource use. Unfortunately, conventional agriculture comes with an intensified rate of land use and reliance on chemical intervention to boost agricultural production in order to satisfy the rising human population and rising nutritional requirements, a practice that often compromises health values (Tal, 2018). On the other hand, modern agriculture is characterized by sustainable farming techniques including the use of improved varieties, preservation, and interconnection in farming systems. To keep this system running efficiently, agriculture must be practiced sustainably. Sustainability in agriculture has been defined as “the successful management of resources for agriculture to satisfy changing human needs while maintaining or enhancing the natural resource base and avoiding environmental degradation” (Sivakumar et al., 2000; O’Neill et al., 2018). Conversely, despite its broad approach and consideration of multiple essential factors, modern agriculture is still faced with several challenges ranging from chemical contamination of soils to loss of plant biodiversity. Concomitantly, reduced diversification often results in increased plant pests and diseases, all of which consequently negatively affect plant health and productivity (Mustafa et al., 2019).

Soil is a multifunctional and extremely complex system with multiple interactions of the chemical, physical, and biological processes that supports a multitude of functions, including the delivery of key ecosystem services (Urra et al., 2019a). However, soil nutrients are depleted through crop cultivation, soil erosion, or leaching, and this adversely affects soil quality and crop output, thereby posing a risk to agricultural sustainability and global food security (Tan et al., 2005). Revitalizing depleted agricultural soils with synthetic fertilizer results in temporarily improved yields. More so, in addition to chemical fertilizers being cost-intensive, their excessive usage has been reported to disrupt the environment, subvert the soil ecology, exert deleterious effects on soil microorganisms, contaminate soil water, reduce soil fertility, and sometimes result in deleterious effects on human health (Maheshwari et al., 2012). Hence, the decline in a plant’s nutrient use efficiency initiates especially under intensive farming systems, a decrease in the level of soil organic matter, i.e., a major cause of soil fertility loss. This was demonstrated in a report that more than 50% of the nitrogen fertilizer applied to cropping systems is not absorbed by plants but is lost to the environment as ammonia (NH_3_), nitrate (${\mathrm{NO}}_{3}^{-}$), and nitrous oxide (N_2_O) (Coskun et al., 2017).

Unlike the challenges associated with the use of agrochemicals, organic manure (including both biomass and animal manure) applied as a soil amendment plays an important role in the recycling of soil nutrients and the management of plant health. Apart from the improved nutrient use efficiency, which accounts for increased crop productivity (Lawal and Babalola, 2014; Yadav et al., 2019), organic amendments are an excellent means of maintaining or increasing the organic matter content in agricultural soils while preserving and improving soil fertility (Akanmu et al., 2020). This is achieved through an increase in the soil microbial community and population due to the application of organic amendments which promote soil and plant health. Soil microbes are responsible for the decomposition and recycling of organic manure through the activities of dehydrogenases, cellulases, proteases, β-glucosidases, phosphatases, arylsulfatases, chitinases, and amylases (Sahu et al., 2017). Furthermore, organic manure plays an important role in enhancing the soil structure and its water holding capacity. It also increases the natural suppressiveness of the soil against soil-borne pathogens, facilitates the amelioration of nutrient-depleted soil, and aids in the restoration of soil structure thereby increasing soil biological fertility (Chukwuka et al., 2020).

While organic amendments can contribute significantly to soil and plant wellness, they can be problematic if they are not adequately applied or managed. Thus, high concentrations of different antibiotics have been detected in some animal manures, which has become a major health concern over the past decade (Zhou et al., 2020). In addition, when improperly handled, livestock manure can adversely affect soil microorganisms and ecosystems while polluting rivers and underground water sources (Tian et al., 2021). One of the approaches that has been researched and employed to overcome this problem is the incorporation of biorational measures of plant disease management (Savoia, 2012; Thimmegowda et al., 2012) and agricultural productivity (Franco et al., 2011). Biorational products are derived from natural or biological origins, such as plants, bacteria or fungi, and include biological pesticides as well as products for crop stress management, increased plant physiology advantages, and root development control. Apart from being biologically and cost-effective tools, the strength and importance of the biorational approach in modern agriculture lies in its relative safety to humans, animals, and the environment (Shaltiel-Harpaz et al., 2016). Therefore, there is need to substitute the use of agrochemicals with biorational farming techniques, to sustainably restore soil health, produce safer agricultural products and ensuring farmers’ profitability. To this end, this study discusses the common agricultural practices and the impact of agrochemicals on microbe-soil-plant interactions. The pros and cons of the application of organic amendments were explored, in relation to its stimulation of quorum sensing, and plant resistance to invading diseases, while the biorational approach of plant health and disease management was reviewed.

## Overview of the Damaging Effect in Conventional Agricultural Practices on the Microbial Complex and Soil-Plant Interaction

### Agricultural Practices: Impacts of Soil Management on Plant Health

Agriculture is a major source of livelihood for many people across the globe. However, despite its huge contribution to the sustenance of humankind, most current agricultural practices negatively impact the environment. Unfortunately, even newer technological developments adversely impact the environment. Furthermore, inappropriate agricultural management practices often alter the natural function of the ecosystem by causing changes in the microbial composition or reduction in the biodiversity of the ecosystem.

One of the most common farming practices is mono-cropping. It, however, negatively affects the microbial landscape of soil, leading to a reduction in the abundance of beneficial microorganisms and causing poor plant growth (Zhao et al., 2018). Tillage is another common agricultural practice; tillage operation is targeted toward loosening the soil, which subsequently decreases soil bulk density and increases soil porosity (Mehra et al., 2018).

If the interactions between soil and the equipment used in farming operations are not appropriately considered, it can result in soil compaction, smearing, and erosion (Mehra et al., 2018; Nweke, 2018). Soil compaction is caused by the heavy farm machinery used for tilling the soil when it is still wet. This has become more problematic over time because most recently developed farm equipment is increasingly heavy. Soil compaction, deep root removal, tillage, application of synthetic fertilizers, insecticides, fungicides, and herbicides all have adverse impacts on the soil. Furthermore, these treatments cause a reduction of the soil air pore size, soil drainage, populations of beneficial worms, insects, bacteria, and mycorrhiza, while providing an enabling environment for various species of pests to thrive with little natural competition (Briones and Schmidt, 2017). Previous studies indicated that soil compaction, which caused a 13–36% reduction in soil aeration (Shah et al., 2017), resulted in a reduction in the microbial biomass nitrogen and microbial biomass carbon. An additional study affirmed that soil compaction results in microbial biomass reduction, poor water absorption, and poor aeration, all of which are often responsible for a reduced crop yield and stunted development of plant roots (Nawaz et al., 2013).

### Impact of Inadequate Chemical Use on Microbial Complex and Soil-Plant Interaction

Aside from the undesirable impact of some agricultural practices that characterize conventional farming, excessive use of agrochemicals mainly enhances the nutritional contents of soil, and mitigating diseases and pests of crops constitute a major challenge to soil health and food safety (Mandal et al., 2020). Also, the significant farm inputs required by this intensive agricultural practice leads to pressure on the environment, whereas agricultural practices that depend on large applications of agrochemicals to repel or control unwanted plants reduce the ability of the ecosystem to attain safe and sufficient yields. More importantly, contamination of surface waters and groundwater, eutrophication, and degradation of soil quality is some of the attributable risks to the use of toxic chemicals on farmland (Khan et al., 2018). In one study, an increase in chemical contamination of nearby water bodies, a rise in the level of greenhouse gases in the atmosphere, and an increased incidence of pathogens was highlighted as some of the detrimental effects of inadequate agricultural practices (Alegbeleye et al., 2018). The list of agrochemical used in cropping agriculture is considerable, consisting of fertilizers, liming, and acidifying agents that alter the pH, soil conditioners, pesticides, and herbicides among others (Ying et al., 2017).

Pesticides are one of the most regularly utilized chemical substances in agriculture. A pesticide is a chemical or combination of substances applied to prevent, extinguish, or repel pests or control plants. Fungicides, nematicides, insecticides, herbicides, molluscicides, and rodenticides are only a few of the examples of these chemicals. Since, insects pests and diseases drastically lower the quality and size of harvestable crops, pesticides are therefore an important part of the plant protection process (Aktar et al., 2009). The primary benefit that makes many farmers find the use of pesticides as an unavoidable aspect of farming is improved crop quality and yield, while the secondary benefits entail food security, the reduced international spread of plant disease, and decreased waste of resources, such as crops, land, water, and time among others. According to one report, an investment of $10 billion in pesticides has been estimated to save up to $40 billion in yearly crop losses (Pimentel and Burgess, 2014). However, despite its importance, a debate over pesticide use and abuse has erupted. Earlier studies have revealed that some commonly used pesticides can cause a decrease in the microbial population and macrofauna diversity in the soil (Rodríguez-Eugenio et al., 2018). In addition, pesticide application alters soil microbiology and decreases the frequency of nitrogen-fixing microorganisms which significantly contribute to soil health and fertility. Soil fumigation has been found to kill most microorganisms inhabiting the soil, including both pathogenic and beneficial organisms. Besides the toxicity of fumigants to soil organisms, they also may be toxic to humans following their application. Studies have reported fumigant accumulation as often exceeding the legal limits in a grape farm (Rodríguez-Eugenio et al., 2018). Despite their efficacy in disease management, pesticides can be harmful to the environment when used in excessive quantities, and their use often results in the development of resistance among the targeted species. To prevent pesticide contamination (and the damage it causes) in our ecosystem, cleaner, non-chemical pest control (including weed control) approaches need to be embraced (Aktar et al., 2009).

Fertilizer application is another major source of chemical use in agriculture. Since its discovery, chemical fertilizers have been widely embraced by crop growers and have played a significant role in enhancing crop productivity. Some of the characteristics that make chemical fertilizers ideal and preferred options to farmers include their rapid plant growth potentials. They also contain the soil nutrient requirement; nitrogen, phosphorus, and potassium in a ready-to-use form and can be modified in the appropriate proportions to help particular soil and crop types achieve the desired higher yields (Liu et al., 2020a). Apart from their efficacy in small amounts, they are convenient to use, whereas the use of chemical fertilizers also comes with shortcomings, such as nutrient runoff from farms which adversely affects the land ecosystems. Some synthetic nitrogen fertilizer has been implicated in the acidification of the soil which can impair the growth of some plants (Neina, 2019), reduce the microbial diversity (fungi, bacteria, archaea, etc.) of the soil, and can shift the soil microbial composition to favor pathogenic strains (Zhou et al., 2017). In addition, the use of synthetic fertilizers in agriculture can cause water pollution and contribute to climate change *via* the production of N_2_O, thereby resulting in algal blooms (Clark and Tilman, 2017). Another major detrimental effect is caused by the excessive use of chemical fertilizer which can lead to the build-up of salts in the soil and the accumulation of nitrate. This is one of the causes of water pollution with harmful effects on humans, and heavy metal contamination (Rodríguez-Eugenio et al., 2018).

## The Pros and Cons of Organic Amendments in the Management of Soil and Plant Health

One of the greatest potential problems of the twenty-first century is the need to preserve soil quality (Scotti et al., 2015). Soil is a highly dynamic natural system that is made up of four primary components, namely: organic matter, mineral matter, water, and air, all of which offer vital ecosystem services for humanity’s survival. Hence, organic soil amendments are considered a potential tool in maintaining soil and plant health through enrichment and maintenance of soil organic matter, which serves as the reservoir of nutrients for plants and beneficial soil microbes. Despite its importance, the application of organic amendments may also have harmful effects on the soil biome and plant health and could constitute a health hazard in some instances, if adequate caution is not adhered to. However, the use of organic soil amendments has taken a back seat while synthetic fertilizers, herbicides, pesticides, and mechanical tillage have become more prioritized in contemporary agriculture.

### The Benefits of Organic Amendments

Organic amendments are natural fertilizers derived from plants and animals. They enrich the soil with the essential carbonic compounds required for plant development while raising the organic matter content and stimulating microorganism reproduction. They also alter the physical and chemical characteristics of the soil (Urra et al., 2019a; Ding et al., 2020), for instance, they increase the soil moisture retention, infiltration rate, structure, aggregate stability, hydraulic conductivity, porosity, nutrient retention, and a reduction in the soil’s bulk density. An increased soil porosity has also been reported to increase the moisture content, infiltration rate, plant root penetration, and nutrient uptake while reducing soil crusting and bulk density (Jien, 2019; Kranz et al., 2020). Organic amendments contain essential micro and macronutrients that are needed for plant growth. They also help to regulate the soil pH and to increase the soil organic matter and the soil’s cation exchange capacity (Abbott et al., 2018). They also play an important role in carbon sequestration, thus mitigating the effects of climate change. Sources of organic amendments include agricultural wastes, i.e., plant biomass and livestock manure, industrial wastes, and municipal sludge. These organic amendments include as: animal manures, green manures, composts, biochar, crop residues, cover crops, and straws among others, all of which are utilized to improve soil and plant health, resulting in sustainable agriculture (Celestina et al., 2019). Unfortunately, synthetic fertilizers, herbicides, pesticides, and mechanical tillage have become more prioritized in contemporary agriculture.

### The Neutral to Detrimental Effects of Organic Amendments

Few instances have been reported where the application of organic amendments did not produce any significant impact on plant growth and disease management compared to the untreated samples. This is evident in the study of Liu et al. (2013) where no significant difference was recorded in the wheat biomass of biochar treated, and the untreated samples, although the result was justified by the interactions of factors, such as soil type, crop species, and environmental condition. Similarly, biosolids treatment of corn produced no significant difference in the plant biomass, compared to the untreated as reported by Lin et al. (2018), while Rady et al. (2016) also demonstrated the non-significance of organo-mineral compost applied at 20–30 ton h^−1^ on the growth of common bean. However, excessive application of organic amendments (e.g., compost) has been reported to result in environmental pollution, such as water pollution (through leaching or runoff), eutrophication, emission of greenhouse gases, heavy metal pollution, persistent organic pollution, and nutrient immobilization, and it could also render the soil acidic or saline (Ozores-Hampton, 2017). In addition, organic amendments that originate from human and animal sources could contain pathogens and antibiotic-resistant genes that are hazardous to human health (De Corato, 2020a).

The existence of dangerous contaminants and chemicals may also pose a threat to the environment when organic amendments made from municipal wastes, pharmaceuticals, and industrial sources are applied (Epelde et al., 2018). This includes the presence of halogenated hydrocarbons, phthalates, heavy metals, pesticides, and food-borne pathogens (e.g., *Salmonella* spp. and *Escherichia coli*; Epelde et al., 2018). Furthermore, micro-plastics have been found in the environment when organic amendments from sewage sludge are applied (Watteau et al., 2018), while the use of organic manure from animal wastes has been reported to often contain harmful microorganisms, antibiotics, and pharmaceutical residues; the antibiotics could result in the proliferation of antibiotic-resistant bacteria in farm soils. Other studies have shown that antibiotics, such as tetracyclines, can remain in the soil for several years and can be absorbed by the crops (Rodríguez-Eugenio et al., 2018). The use of animal wastes from industrial animals can lead to heavy metal accumulation from the metals present in their feeds; some of these metals include lead, copper, and zinc (Wuana and Okieimen, 2011).

### Overview of Some Common Organic Soil Amendments in Use

Organic soil amendments modify the soil structure, thereby allowing a more efficient absorption and retention of water and nutrients in the soil. Some of the common organic amendments in use are compost, crop residues, cover crops, livestock manures, sewage sludge, and biochar among others.

#### Compost

Compost is a stable, hummus-like end-product obtained from the breakdown of organic materials under regulated aerobic conditions. They are produced from a variety of organic resources including animal manures, crop residues, sewage sludge, and municipal solid waste (Azim et al., 2018). Composting is a multi-step process with appropriate measurements of air, water, carbon, and nitrogen sources. Composted amendments enhance soil aeration, porosity, aggregate stability, water holding capacity, and nutrient availability while promoting microbial activity in agricultural soils. Evidence had shown that the employment of composted amendments is an effective means of managing soil and plant pathogens (Joos et al., 2020). Compost has long-term effects on the soil. However, the process of composting could predispose humans to aspergillosis, tetanus, paronychia, and histoplasmosis. More so, phytotoxicity was well reported when compost from market waste, cattle dung, and fresh grass was applied in different concentrations on cowpea (Obuotor et al., 2017).

#### Livestock Manure/Waste

Animal manure refers to the solid, semisolid, and liquid waste products produced by animals raised to produce milk, meat, eggs, and other agricultural goods for human use (Urra et al., 2019b). In medium to long-term application durations, animal manure can enhance soil organic matter. As a result, animal manure aids in soil bulk density and compaction reduction, as well as soil aggregate stability, water infiltration, and retention. Manure-based supplements can increase soil microbial activity and biomass while also changing the makeup and diversity of soil microbes (Zhang et al., 2018). However, animal manure may contain resident pathogens, and this can constitute dangers to human health especially in the case of antimicrobial resistance microorganisms (Watts et al., 2017).

#### Crop Residues

Crop residues are materials left on cultivated land after the crop has been harvested. Crop residues can improve soil structure, increase organic matter content in the soil, reduce evaporation, and help fix CO_2_ in the soil (Nikolić et al., 2021). These plant-based amendments are considered the greatest source of soil organic matter for agricultural soils. They can provide protection against soil erosion, suppress weeds, improve soil physicochemical and biological properties, and enhance soil fertility (Wang et al., 2017). Whereas, although covering the soil with agricultural residues has been shown to reduce weed emergence, there is still a paucity of evidence on the weed species-specific emergence response to different types and quantities of residues (Wang et al., 2017).

#### Biochar

This is the solid product obtained from the thermochemical breakdown of biomass (plants and animal origins) under oxygen-limited conditions within a moderate temperature of 350–700°C (Chukwuka et al., 2020). It is produced from a wide range of feedstocks, including wood, plant waste, and even manure. The qualities that qualify biochars’ use as an organic amendment in agriculture entail its high carbon content, stability, surface area, and high pH. Biochar has a unique capacity to build soil, conserve water, create renewable energy, and store carbon; it has the potential to be a significant tool for the agricultural industry, especially with its sterile nature which does not harbor resident pathogens. Its efficacy against some plant diseases has been investigated (Akanmu et al., 2020). A study by Liu et al. (2020b) reported a decrease in the abundance of amoeba, fungivores, herbivores, and bacterivores nematodes when biochar was applied as soil amendments. However, some of the drawbacks associated with the inappropriate production process or handling of biochar could be inhibition of germination, and possible yield decline. The research carried out by Wu et al. (2021) also indicated that the application of biochar leads to a decrease in the soil’s organic carbon and total nitrogen.

### The Role of Beneficial Microorganisms in the Performance of Organic Amendments

Bacteria and fungi from organic amendment origin play significant roles in nitrogen fixation and phosphorus, iron, and potassium mobilization (Glick, 2012, 2015). Moreover, the hyphal growth of fungi enhances soil aggregation and aggregate stability (Urra et al., 2019a). *Pseudomonas aeruginosa*, *Alcaligenes faecalis*, and *Proteus penneri* from organic amendments were reported to release metabolites that cause an increase in the leaf area, soil moisture content, root length, plant biomass, and shoot length of plants (Naseem et al., 2018). *Bacillus* species found in organic amendment can reduce saline stress on wheat by producing exopolysaccharides and degrading 1-aminocyclopropane-1-carboxylic acid (ACC; Din et al., 2019).

A large number of different types of bacteria, all expressing ACC deaminase, can significantly decrease the negative effects of a wide range of abiotic and biotic stresses (Glick, 2004; Phour et al., 2020). Microbes mitigate plant diseases by inducing plant resistance (Vannier et al., 2019), competing for space and nutrients (Shafi et al., 2017), hyper parasitism (invasion and destruction of fungal spores and mycelium as well as bacterial cell destruction; Ghorbanpour et al., 2018), and antibiosis (Chandra and Kumar, 2017). The application of organic amendments to soil also bio-remediates the soil from pollutants (Glick, 2003). It immobilizes heavy metals by forming metallo-humic complexes which are chemically stable (Alam et al., 2020), reducing the contaminants to a less toxic form, or increasing the soil’s pH, thereby making the metals less available for plant uptake (Lacalle et al., 2020).

## Biorational Approach to Sustainable Agricultural Practice

Humans need to eat well for sustainable health living. Conversely, the challenge of food safety and security occurs as a result of the inefficiency of farm input, farm practices including disease and insect pest infestation ravaging farm crops resulting in low yields (Dubey et al., 2019; Adeleke and Babalola, 2020b). Thus, it is imperative to boost agricultural production using up-to-date technology and human involvement along the food production value chain. The continuous or indiscriminate use of agrochemicals in enhancing crop yield and control of pest infestations on crops has led to the accumulation of pesticide residues on crops, new pest insurgence, development of resistant biotype, and outbreaks of secondary pests (Thakur et al., 2020). Efforts through research have devised ecologically, friendly agriculture devoid of chemical fertilizer application to boost crop production. To circumvent crop pest and disease infestation threats and ensure a safe environment for optimum crop yield, it is important to explore promising alternative measures in the control of crop enemies including a biorational approach to the practice of sustainable agriculture.

The word “biorational” is derived from the words “biological” and “rational” (Kapoor and Sharma, 2020). A biorational approach is a farming system which incorporates ecologically and economically sound agricultural practices in its operations, such as cover crops, crop rotations, and no-till which supports reduced inputs for crop production. This system leverages the advantages of both conventional and organic agriculture to produce more sustainable farming operations with minimal or no adverse effects. The biorational approaches are soft on the beneficial organisms and cause minimal disruption of the natural origin of the microbial biome. It is a dynamic approach to agriculture with huge potential for enhancing crop productivity, managing pest and diseases, and ensuring healthy soil and plants (Gogi et al., 2017). Although, researchers have given more attention to its role as a biopesticide, the potentials of biorational products for crop stress control, improved plant physiology benefits, soil health, irrigation efficiency, and root development management have been reported.

As “third-generation pesticides,” biorational pesticides which mostly include minimum-risk pesticides, organic pesticides, and biological control agents are made from natural sources and offer little or no risk to the environment or beneficial creature. The microbial pesticides *Bacillus thuringiensis*, *oxadiazines*, *phenylpyrazoles*, *avermectins spinosyns*, *pyrroles*, and other pest growth regulators are some of the examples of biorational pesticides. Pests with a narrower target range tend to have a more specialized mode of action (Horowitz and Ishaaya, 2004).

**Figure 1:**
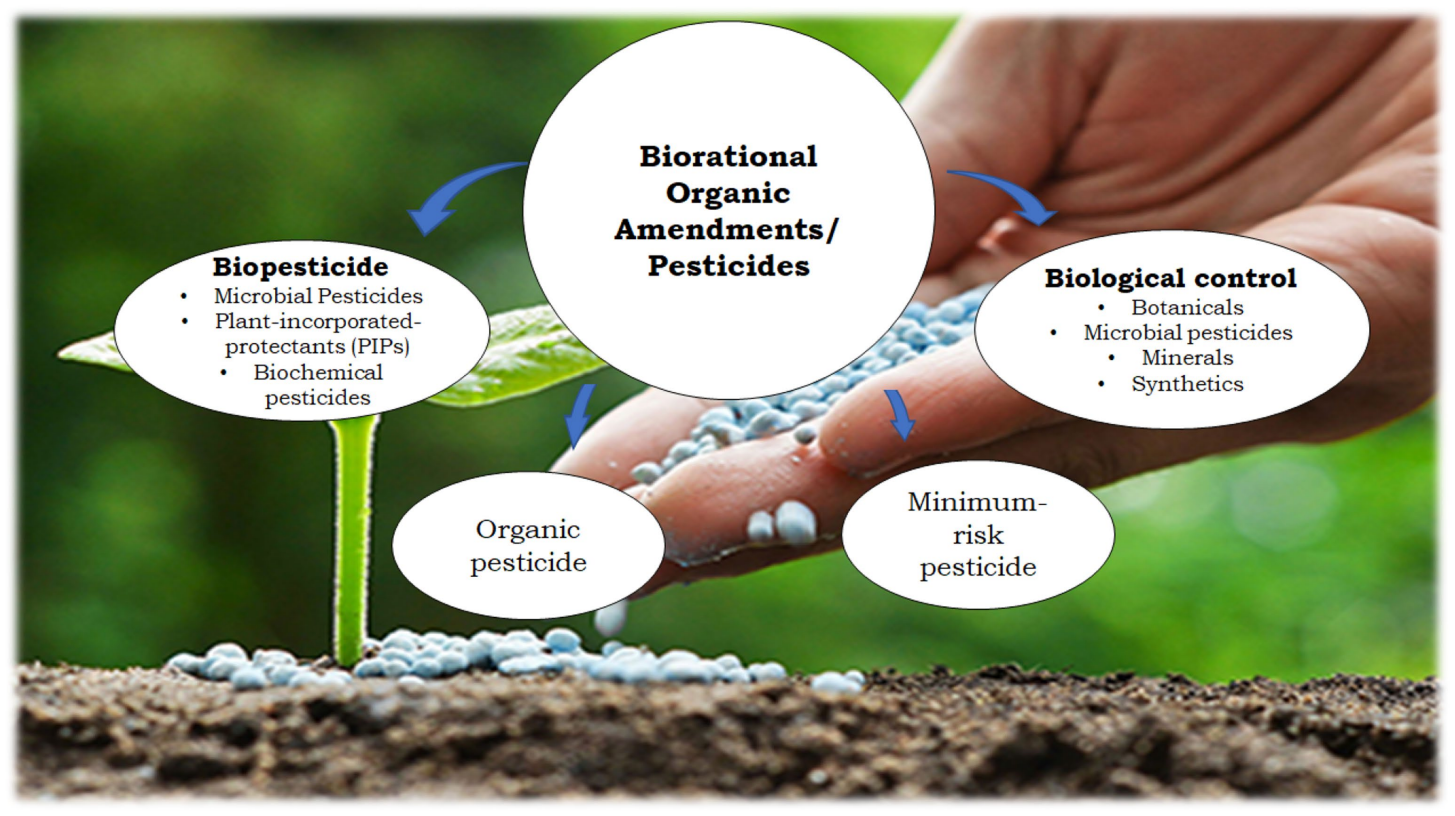
Biorational use of organic amendments in agriculture.

Biopesticides are derived from nature through elements within natural ecosystems, such as animals, plants, microbes, and certain minerals which are subjected to registration regulations. These ecofriendly pesticides have been used effectively for decades in both organic and conventional agriculture, and are relatively safe and non-toxic to humans, except in a few cases of environmental side effects. They consist of botanicals, microbial pesticides, minerals, and synthetics, which are grouped into categories that include biopesticides, organic pesticides, minimum-risk pesticides, and biological control agents (Abrol and Shankar, 2017; Figure 1).

Control of crop pests for improving crop production using bioinoculants, resistant cultivars, biological, cultural, mechanical, sex pheromone, mating disruption, intercropping, and crop rotation has all been employed (Moussa et al., 2018). Nevertheless, using a biorational approach in integrated crop management (ICM) for plant immunity and control of plant pests is promising as an optional technique for organic amendment in crop production (Mukherjee et al., 2019).

Recent efforts toward ensuring food production for the world population in a more sustainable manner by employing a biorational approach need more focus. The inclusion of bioinoculants (biofertilizers and bio pesticides) which are tailored formulations of individual strains or consortia of known microorganisms that promotes plant development when applied either as seed coating or directly to the soil (Owen et al., 2015) can ensure sustainable agriculture depending on the prevailing environmental conditions. However, crop growth in marginal soil is faced with numerous environmental constraints that affect plant growth and physiological performance (Singh et al., 2016). For example, the insect pests *Helicoverpa armigera*, *Maruca testulalis*, *Lampides boeticus*, *Exelastis atomosa*, *Melanagromyza obtuse*, *H. armigera*, and *Grapholita critica* negatively affect plant development and cause loss of crop yield (Sayeeda et al., 2021). The use of pesticides in controlling pests by pest managers is an optional approach in conventional agricultural practice. The rate of pesticide application determines the level of toxicity to the ecosystem, as many pests resist a majority of pesticides. It is thus paramount to acquire tools to implement an environmentally friendly and low-cost approach in ICM using microorganisms (Igiehon and Babalola, 2017).

Bioinoculants function in the protection of plants, promote plant growth, and ensure the bioavailability of nutrients for plant use. A small dose of a biofertilizer is sufficient to produce an expected outcome compared to multiple applications of chemical fertilizer (Romero-Perdomo et al., 2017).

Biorational pesticides from microorganisms are less toxic, inexpensive, easily degradable, and easy to develop and should therefore be desirable by researchers and farm managers as organic amendments in improving crop production (Hossain et al., 2020). Common microbial products used as biopesticides in the control of tomato pests (*Tuta absoluta*) include spinosad from *B. thuringiensis*, azadirachtin from *Metarhizium anisopliae*, and Balsamo from *Beauveria bassiana* (Moussa et al., 2018). Also, from a plant source, the inclusion of kairomones (chemicals emitted by an organism that mediates interspecific interactions) in the control of *Bactrocera dorsalis* (oriental fruit flies) has been documented (Hossain et al., 2020). Other examples include bioinsecticides (*B. thuringiensis*), biofungicides (*Trichoderma* spp.), and bioherbicides (*Phytophthora* spp.) (Adeniji and Babalola, 2018; Olowe et al., 2020).

The biorational approach is, however, not limited to plant disease control but also contributes directly to plant health. The use of bioinoculants instead of agrochemicals helps amend soils that are deficient in essential nutrients (Igiehon and Babalola, 2017; Adeniji et al., 2021).

Taking advantage of modern agricultural practice over traditional methods can ensure sustainable agriculture with diverse environmental benefits. The importance of microbes in agriculture has been studied. Plant growth-promoting microbes significantly promote plant growth and commercialization of microbial products as principal stimulants to amend the soil for sustainable soil health in enhancing plant growth can further improve agricultural productivity (Reed and Glick, 2013; Santoyo et al., 2016). The use of microorganisms as a source of bioinoculants plays a significant role in enhancing plant growth and control of phytopathogens (Sobowale et al., 2010). Important agricultural microbes exhibit multifunctional attributes in that they are promoting plant growth including solubilization of soil mineral elements, such as phosphorus, potassium, and zinc, nitrogen fixation, enzymes, and siderophore production (Adeleke and Babalola, 2020a). These attributes and their survival under different stress conditions make beneficial microbes a suitable bioresource in organic farming (Kour et al., 2020). The application of biofertilizers for improved crop production has received a boost in recent times (Lawal and Babalola, 2014; Igiehon et al., 2019). Therefore, it is imperative to substitute agrochemicals with bioinoculants. In addition, crop management and pest control can be achieved by incorporating viable microorganisms as microbial inoculants. Information regarding the use of microbial inoculants can ensure sustainable agriculture depending on the level of their consistency (Table 1).

**Table 1:** Biorational management of unhealthy soil and plants.

Properties	Symptoms/causes of unhealthy soil and plants	Types of biorational organic amendment to use	Impact of Organic Amendment	References
Physical	Silt or compaction of soils	Organic amendments (from manure and plant-based), biochar, compost, biofertilizer, and plant-microbe rhizosphere engineering	Soil aeration, environment, and ecosystem restoration. Promoting plant and soil wellness	Scotti et al., 2015; Igiehon and Babalola, 2017; Abbott et al., 2018; Enebe and Babalola, 2019; Jien, 2019; Cardinale et al., 2020; Adeniji et al., 2021
	Mechanical damage			
	Leaching/erosion			
	High or low temperatures			
	Reduced oxygen availability			
	Drought/excess soil moisture			
	Limited light			
	Air pollution			
	Non-absorbent of water			
Chemical	Pesticide toxicity	Biochar, compost, biochemical pesticides, microbial pesticides, and minimum-risk pesticide	Remediation of contaminated soils. Reduces the environmental risks associated with the use of chemical fertilizers and pesticides	Jien, 2019; Akanmu et al., 2020; Alam et al., 2020; Chukwuka et al., 2020
	contaminated soils			
	Presence of heavy metal			
	Soil salinity and sodicity			
	Soil pH extremes			
	Carbon sequestration			
Biological	Bacteria	Biopesticides, organic pesticides, minimum-risk pesticides, and biological control agents. Biorational adoption of Integrated Pest Management System	Ecofriendly pesticides effectively managed soil/ plant diseases, they are relatively safe and non-toxic to humans	Gressel et al., 2007a,b; Abrol and Shankar, 2017; Bonanomi et al., 2018; Enebe and Babalola, 2019; Herren et al., 2020; Ojuederie et al., 2021
	Fungi			
	Nematodes			
	Viruses			
	Parasitic higher plants			
	Protozoa			
	Insects			
	Mites			
	Others			

## Role of Organic Amendment in Stimulating Quorum Sensing in the Beneficial Microbe

Microbe-microbe and plant-microbe communication occur *via* the exchange of a wide range of signal molecules, many of which have a significant effect on microbial behavior. One of these signaling mechanisms in bacteria controls the phenomenon of quorum sensing, which is the regulation of gene expression in response to cell population density. Quorum sensing regulation is used by both plants’ pathogenic and beneficial bacteria to control their phenotypes which include the production of extracellular enzymes/metabolites/factors, movement, and biofilm formation (Von Bodman et al., 2003; Schikora et al., 2016). For example, quorum sensing plays an important role in enhancing beneficial rhizosphere communities and aiding the development of symbiotic relationships of bacteria with plants. In parallel, several bacterial pathogens use quorum sensing to coordinate and synchronize bacterial behavior in plant attack and expression of virulence factors. Interfering with quorum sensing can therefore have either beneficial or detrimental effects concerning plant-bacteria interactions depending upon the bacteria involved. Controlling these cell–cell signaling systems among microbes could be a way of defending against root pathogens; however, disruption of signaling could also affect plant-bacteria beneficial interactions. In addition to quorum sensing, several interkingdom signaling systems take place between microbes and plants to allow for the formation of beneficial microbial plant-associated communities (Venturi and Keel, 2016). Similarly, signaling that occurs between plants and pathogenic bacteria plays an important role in the resistance and susceptibility of host plants to the pathogen (Venturi and Fuqua, 2013).

Soil management strategies consisting of the provision of organic fertilizers most likely significantly influence these signaling mechanisms among microbes *via* the degradation or bioavailability of signaling molecules. For example, it has been reported that plant-rhizobia communication *via* flavonoids is affected by soil organic matter since it interferes with plant-bacteria signaling by affecting flavonoid presence hence reducing the formation of root nodules (Del Valle et al., 2020). Another recent study has indicated that the addition of organic fertilizer had an impact and stimulated the presence of the *Pseudomonas* spp. bacterial population which resulted in a more disease-suppressive soil (Tao et al., 2020). It is not yet known how bio-organic fertilizers increase the population of plant-beneficial *Pseudomonas* spp.; it cannot be excluded that it is *via* the stimulation of quorum sensing systems that allow *Pseudomonas* spp. to be more concentrated compete better for nutrients. In summary, quorum sensing is important for microbes that live in soils. With this system, it is essential to have a wide diversity of single-celled microbes in soils to work together collectively and function as a coordinated multi-celled super-organism. There is little detailed information regarding quorum sensing, and hence, there is an urgent need to understand these mechanisms in soils and whether they can be affected by organic amendments.

## Role of Organic Amendments in Stimulating Isr and Sar in Plants Against Invading Diseases

Organic amendments play a significant role in the induction of disease resistance in plants, a phenomenon that has been related to variations in the signaling pathways as systemic-acquired resistance (SAR) and induced systemic resistance (ISR). These terms are considered appropriate only when there is consistent observation of biologically relevant resistance (De Kesel et al., 2021). While ISR is a mechanism activated by infection, SAR is activated in plant after its exposure to elicitors from virulent, avirulent microorganisms or artificial chemical stimuli (Kamle et al., 2020).

Organic amendments and advantageous microbes have been researched and utilized globally, yet the progression of successful disease management has been constrained by poor incorporation of research and methodical approaches (Bonanomi et al., 2018). In some instances, organic amendments have been described as ineffectual for disease control and they occasionally boost the intensity of plant diseases. A meta-analysis of organic amendments considering 2,423 studies reported that 45% of OAs were suppressive and 35% had little influence, while 20% brought about an increase in the occurrence of diseases. Thus, it is imperative to have a more detailed understanding of the mechanisms controlling organic amendment-based disease suppressiveness (Bonanomi et al., 2018).

Many microbiotas coexist within the soil, utilizing a wide range of organic carbon sources. A paucity of these organic carbon sources can cause severe competition among microorganisms. Organic amendments can relieve organic carbon starvation, subsequently modulating the interactions and stability of microbial communities since they provide an expanded food base (Bonanomi et al., 2018). The effect of organic amendments to reduce the number of pathogens and their virulence in the soil microbiome has been investigated since the 1980s (De Corato, 2020b).

The introduction of organic amendments into the soil causes a disturbance of the microbiota giving rise to a change in the structure of the network of microbial communities specifying suppression against phytopathogens and the diseases that they trigger. Significant targets have been achieved by stimulating the explicit intrusion of the soil microbiome through agricultural procedures centered on altering and shaping the network of microbial communities for intensifying the innate suppressiveness of soil (De Corato, 2020b). Organic amendments can be tailored to induce substantial shifts of the soil microbiome to the desired favorable consortia which enables a sustainable cropping system without further input of agrochemicals (Vacheron et al., 2015; Bonanomi et al., 2020; De Corato, 2020b). The prospect of employing organic amendments for disease suppression against oomycetes, *Pythium* spp., *Phytophthora* spp., *Fusarium oxysporum*, and *Rhizoctonia solani* (Jambhulkar et al., 2015) among others is typically pathogen-specific and differs extensively among the pathogen types (Bonanomi et al., 2020).

Several mechanisms have been suggested to explain the mechanism(s) of organic amendments disease suppressiveness. In this regard, the activity of soil microorganisms is wholly implicated in this process as with other soil activities (Amoo and Babalola, 2017; Bonanomi et al., 2018). The direct instigation of defense or priming of cells by organic amendments gives rise to greater elicitation of defenses after a pathogen infestation is triggered. A physiological condition where the intrinsic defenses of plants are enhanced against disease-causing agents on exposure to abiotic or biotic stimuli is recognized as induced resistance. ISR and SAR are the two main types of induced resistance. Organic amendment-mediated induced resistance can be stimulated either by biological or chemical processes or both. Through the modulation of beneficial microbial communities, organic amendments, such as compost and biochar, can induce plant defense responses (Bonanomi et al., 2018), although the amendments may in some cases trigger other negative or neutral effects (De Tender et al., 2016).

The induction of systemic disease resistance is well known to be triggered by chemical compounds, some originating from organic amendments and others secreted by non-pathogenic microorganisms linked to plants (Deng et al., 2020). In specific pathosystems, some organic amendments induce systemic resistance and encourage transcriptional alterations in plant protection-associated pathways (Enebe and Babalola, 2019; Gupta and Bar, 2020). When plant roots are colonized by specific strains of plant growth-promoting bacteria, ISR, which is mediated by a jasmonate (JA)- and ethylene (ET)-sensitive pathway, develops. The induction of defense responses by ISR is not direct; instead, a physiological state allowing plants to respond more effectively is fostered. This is known as a priming effect. SAR is, however, mediated by a salicylic acid (SA)-dependent pathway and is stimulated by treatment with an assortment of agents. It is triggered after localized attacks by pathogens (Abdul Malik et al., 2020; Maithani et al., 2021).

## Conclusion

Contrary to the side effects of excessive use of agrochemicals in managing pests and diseases but which constitute a threat to biodiversity conservation and the extinction of beneficial fauna, biorational approach to agriculture provides a safe, ecofriendly, and sustainable alternative with minimal or no adverse impacts. The biorational farming system use of organic amendments entails the utilization of naturally produced, registered, and regulated products that are target-specific, promote plant growth and health and are safe for human use. Such products have overcome the detrimental effects attributable to raw organic amendments. A biorational approach has been explored in the study of insects, but there exists a paucity of research on its use against plant pathogens. The biorational products are potential candidates of a sustainable integrated pest management program. It is therefore necessary to focus on the development of biopesticides and biofertilizers suitable for registration and production at an industrial level to facilitate their availability to the farmers, who are the ultimate end users.

## Author Contributions

All authors listed have made a substantial, direct and intellectual contribution to the work, and approved it for publication.

## Conflict of Interest

The authors declare that the research was conducted in the absence of any commercial or financial relationships that could be construed as a potential conflict of interest.

## Publisher’s Note

All claims expressed in this article are solely those of the authors and do not necessarily represent those of their affiliated organizations, or those of the publisher, the editors and the reviewers. Any product that may be evaluated in this article, or claim that may be made by its manufacturer, is not guaranteed or endorsed by the publisher.
